# The Role and Mechanism of Deubiquitinase USP7 in Tumor-Associated Inflammation

**DOI:** 10.3390/biomedicines12122734

**Published:** 2024-11-29

**Authors:** Luhong Wang, Yong Zhang, Tao Yu, Huijian Wu

**Affiliations:** 1 Cancer Hospital Affiliated to Dalian University of Technology, Shenyang 110042, China; wangluh@dlut.edu.cn (L.W.); zhangyong@cancerhosp-ln-cmu.com (Y.Z.); 2 Dalian Key Laboratory of Protein Modification and Disease, Faculty of Medicine, School of Biological Engineering, Dalian University of Technology, Dalian 116024, China

**Keywords:** USP7, deubiquitination, inflammatory, cancer

## Abstract

Deubiquitinating enzymes are a class of proteases that remove ubiquitin tags from proteins, thereby controlling protein stability and function. Tumor inflammation arises from interactions between tumor cells and their microenvironment, which trigger an inflammatory response. The deubiquitinating enzyme USP7 plays a central role in this process. Research suggests that USP7 may modulate various signaling pathways related to inflammatory responses through its deubiquitinating activity, thereby influencing tumor development and progression, including regulating T cell immune activity, improving macrophage anti-tumor activity, and regulating NF-κB signal pathways. Overall, describing the role and mechanism of USP7 in the tumor inflammatory response is of great importance for elucidating the regulatory mechanism of tumor inflammation and developing new therapeutic strategies. This article mainly reviews the structure, function, role, and mechanism of USP7 in the tumor inflammation response.

## 1. Introduction

**Figure 1:**
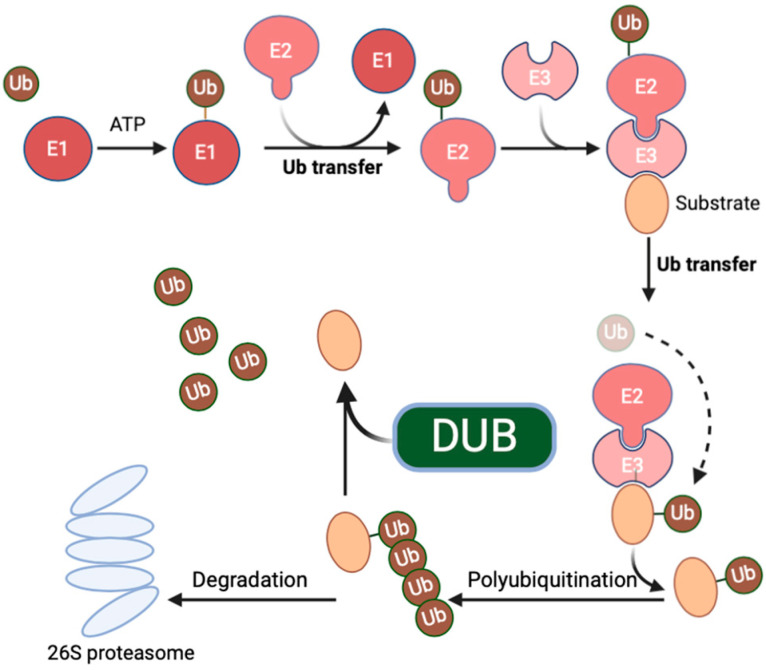
The ubiquitin–proteasome system. Ubiquitination is a multi-step process, which involves the activation of ubiquitin by E1 enzymes, the conjugation of ubiquitin with E2 enzymes, and the conjugation of ubiquitin to substrate proteins via E3 enzymes. Ubiquitination can lead to the degradation of substrate proteins by proteasomes or to their recruitment into multi-protein complexes, depending on the topology of the polyubiquitin chain linkage. Deubiquitinating enzymes remove the Ub moiety from substrate proteins with high specificity and reverse the Ub signal to maintain dynamic cellular ubiquitination. The dashed line represents the process that occurs simultaneously.

Deubiquitinating enzymes (DUBs) belong to a category of enzymes capable of removing ubiquitin tags from proteins. They play crucial roles in various biological processes, including cell signaling, protein homeostasis, and the cell cycle [1] (Figure 1: The ubiquitin-proteasome system). Ubiquitin-specific protease 7 (USP7), a member of the DUB family, exhibits a wide range of substrates, including key proteins involved in tumor suppression, DNA repair, and immune responses [2]. As a result, the role and mechanisms of USP7 in tumor inflammation have become a focal point of recent research. Functionally, USP7 exerts multifaceted regulatory effects in tumor inflammation. On one hand, it can influence the expression of tumor suppressor genes. For example, by modulating the stability of the E3 ligase MDM2, USP7 can indirectly impact the functionality of tumor suppressor genes such as p53. The degradation of MDM2 leads to the stabilization and reactivation of p53, thereby inhibiting tumor growth [3]. On the other hand, USP7 also participates in regulating immune responses [4,5]. Its interactions with immune-related proteins may influence the intensity and duration of inflammatory responses, thereby influencing tumor onset and progression [6].

From a mechanistic perspective, the role of USP7 in tumor inflammation is mainly exerted through its deubiquitinating activity [7]. Ubiquitination is a significant protein modification technique that controls a variety of biological processes in cells by affecting the stability and functionality of proteins [8]. Deubiquitinating enzymes can reverse this process and return proteins to their original state. Within tumor inflammation, USP7 modulates tumor growth and immune responses by removing ubiquitin tags from key proteins, thereby altering their stability and function [4].

It is important to note that the activity of USP7 could be subject to regulation by a multitude of factors. Changes in the intracellular environment and interactions with other enzymes or molecules could potentially impact USP7’s deubiquitinating activity [9]. Consequently, within the realm of tumor inflammation, the operational mechanism of USP7 likely represents a complex and finely tuned network. Further research is required to elucidate its specific modes of regulation and influential factors. USP7 plays a pivotal role in tumor inflammation, with its mechanisms encompassing various facets such as the expression of tumor suppressor genes and the modulation of immune responses. As our understanding of USP7 continues to evolve, we expect to gain a more comprehensive understanding of its role in tumorigenesis and progression, thereby providing new perspectives and strategies for cancer therapy.

## 2. The Structure, Function, and Expression of USP7

### 2.1. Structure and Function of USP7

**Figure 2:**
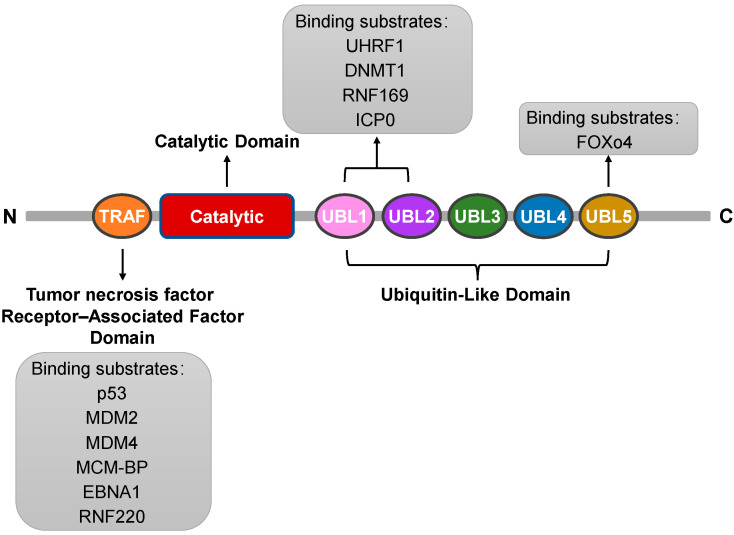
Schematic diagram of USP7 indicating the amino acid positions of each structural domain and each substrate-binding region. The list below the schematic diagram shows the protein substrates known to bind to each substrate binding site.

The USP7 protein is a uniquely structured deubiquitinating enzyme that plays a vital role in the dynamic process of intracellular protein degradation [10]. The protein structure of USP7 can be elucidated from several aspects: First, USP7 consists of a specific sequence of amino acids connected to each other via different chemical bonds, forming a complex three-dimensional structure. It comprises a total of 1102 amino acid residues with a relative molecular mass of 135,000 Da, highlighting its properties as a large protein [2]. Second, from a structural domain perspective, USP7 includes three main domains. The first is the tumor necrosis factor receptor-associated factor (TRAF) domain at the amino terminus (amino acids 53–205), a crucial site for USP7 to recognize and bind various proteins. It can interact directly with substrate proteins such as the tumor suppressor gene p53 and the Mouse Double Minute 2 (MDM2) protein [11,12]. The second is the highly conserved USP domain (amino acids 208–560), which serves as a catalytic center and is essential for the catalytic activity of USP7 [2]. Finally, it includes five C-terminal ubiquitin-like domains (UBL1-5) (amino acids 564–1102), which may be associated with protein–protein interactions and localization [13]. Furthermore, the interplay and relative positioning between these structural domains are also important aspects of the protein structure of USP7. Together, they maintain the stability and functional activity of USP7 through specific spatial arrangements and interactions. In summary, USP7 exhibits complex structural characteristics, which underpin its unique biological functions (Figure 2: Structure of USP7).

**Figure 3:**
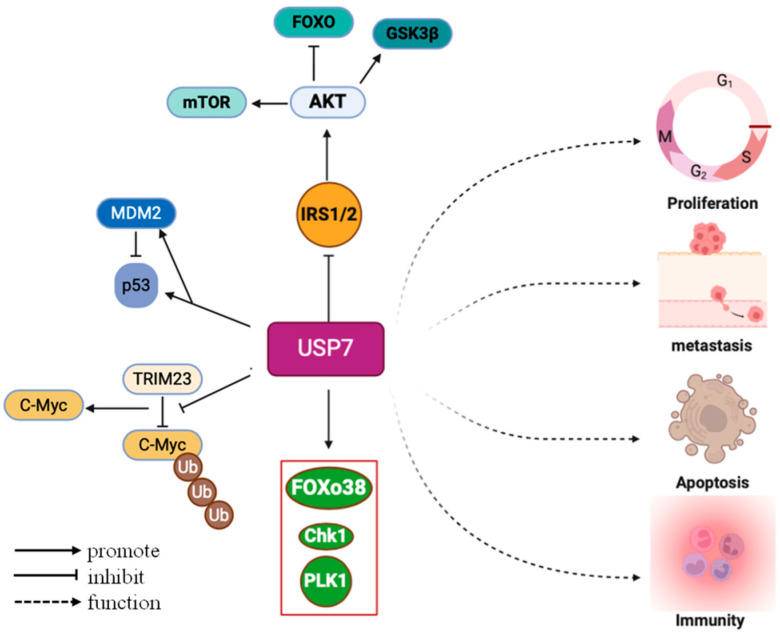
USP7 supports the sustained proliferation of cancer cells and resistance to growth signals. USP7 maintains growth by stabilizing IRS1/IRS2. USP7 regulates cell cycle checkpoints. USP7 controls the stability of P53 and MDM2. The stability of the oncogene C-Myc is also mediated by USP7.

USP7 has a number of complex functions, mainly due to its extensive substrate repertoire, which includes the tumor suppressor gene p53, MDM2, phosphatase and tensin homolog, nuclear transcription factor κB (NF-κB), and inflammasomes [14,15,16,17]. Furthermore, USP7 is involved in numerous aspects such as DNA damage repair, epigenetic regulation, and viral infections [18,19,20]. It is involved in the repair of oxidative DNA damage by modulating chromatin structure and regulating the stability of PCNA via its deubiquitinating function, thereby controlling DNA replication and damage repair processes [21,22]. Depletion of USP7 or FBXO38 can lead to a significant decrease in KIF20B levels and midbody KIF20B, thereby impairing cytoplasmic division [23]. At the same time, USP7 can also influence the stability and distribution of epigenetic markers and thus regulate gene expression patterns. During viral infection, USP7 can regulate the stability and functionality of viral proteins, thereby influencing the replication and infection process of the virus [20] (Figure 3). 

USP7 was initially discovered in 1997 to interact with the early protein of herpes simplex virus, thereby promoting viral growth, highlighting its central role in the viral life cycle [13]. In addition, USP7 also plays a key role in the regulation of neurological diseases, immune responses, the regulation of tumor-related signaling pathways, and the consequences of inflammatory diseases [4,5,24,25,26]. By modulating various substrates, it plays an important role in processes such as cell apoptosis and cell cycle regulation. Research has revealed that USP7 stabilizes c-Myc through ubiquitination, and this function is essential for maintaining neuronal stem cell fate [27] (Figure 3). USP7 is located near the replication body and its activity is essential for maintaining active DNA replication and preventing replication stress independent of PCNA ubiquitination or p53 [21,28]. This indicates that it also plays an indispensable role in the process of DNA replication.

### 2.2. USP7 Expression and Cancer Initiation, Progression, and Drug Resistance

In the field of cancer research, the expression of USP7 has attracted great attention. Broadly speaking, USP7 expression shows an upward trend in various cancer types and is closely associated with cancer onset, progression, and drug resistance [29,30]. First, increased USP7 expression is positively correlated with the severity and poor prognosis of multiple cancers, suggesting a pivotal role for USP7 in cancer progression. USP7 can promote malignant behavior in cancer cells such as proliferation, migration, and invasion. For example, increased USP7 expression in oral squamous cell carcinoma promotes tumor cell proliferation and invasion [31]. In hepatocellular carcinoma (HCC), USP7 overexpression is significantly associated with malignant phenotypes such as tumor enlargement, poor differentiation, and microvascular invasion [32]. Suppression of USP7 expression can inhibit the growth and metastasis of melanoma cells both in vitro and in vivo [33]. The expression patterns and functional differences of USP7 in different cancer types are mainly reflected in its mechanism of action and signaling pathways in tumor progression (Figure 3). We can summarize the following points:

P53 signaling pathway: USP7 stabilizes MDM2 and MDMX through ubiquitination; these are the main negative regulatory factors of p53. In this way, USP7 inhibits p53 degradation and thereby suppresses p53-mediated cell cycle arrest and apoptosis, thereby promoting tumor cell survival and proliferation [3]. In addition, USP7 was found to stabilize LSD1, further inhibit the p53 signaling pathway, and promote tumorigenesis and metastasis of glioblastoma [34].
The PI3K/Akt/FOXO and AMPK signaling pathways: Knocking down or knocking out USP7 can increase the expression of AMPK beta, caspase 7, and PPP2R3A while reducing the expression of ATP6V0 and PEX11B, thereby inhibiting the proliferation of melanoma cells [35].
Wnt/β-catenin signaling pathway: USP7 can directly bind to β-catenin, activating the Wnt/β-catenin signaling pathway and inducing epithelial–mesenchymal transition (EMT), a crucial process in tumor cell invasion and metastasis [36].
PI3K/AKT signaling pathway: Ubiquitin-specific protease 7 promotes osteosarcoma cell metastasis by inducing epithelial–mesenchymal transition [37].
NF-κ B/PD-L1 signaling pathway: USP7 promotes cervical cancer initiation and progression by upregulating EZH2 expression, downregulating TIMP2 expression, and consequently activating the NF-κB/PD-L1 signaling pathway [14].
Insulin/IGF signaling pathway: USP7 is an IRS-1/2 deubiquitinase that establishes a negative feedback loop in insulin/IGF signaling [38].

Second, USP7 may also influence cancer progression by modulating antiapoptotic pathways, including DNA damage signaling, starvation signaling, and oncogene activation signaling. This disrupts the intracellular balance and fosters cancer development. For instance, in breast cancer cells, the dysregulation of USP7 disrupts S-phase-specific DNA repair and exacerbates DNA replication stress, potentially resulting in increased chromosomal instability and reduced overall survival rate [39]. USP7 can reduce the levels of the endonuclease DICER, thereby impairing the response to DNA damage and facilitating cancer progression [40]. USP7 inhibitors can induce apoptosis in glioblastoma cells by promoting the ubiquitination of ARF4 [41].

In addition, USP7 is closely linked to anticancer drug resistance. During chemotherapy, increased USP7 expression may engender drug resistance in cancer cells, thereby diminishing treatment efficacy. Consequently, targeted therapy against USP7 could emerge as a novel strategy to overcome anticancer drug resistance. For instance, USP7 stabilizes KDM5B via the ZBTB16/TOP2A axis, promoting nasopharyngeal carcinoma progression and resistance to cisplatin [42]. USP7 and PLK inhibitors show strong synergistic effects and hold promise for the treatment of paclitaxel-resistant cancers [43]. USP7 maintains the stability of the PLK1 protein through deubiquitination and regulates chromosome alignment during mitosis. When USP7 is inhibited, it can trigger cell apoptosis and arrest the cell cycle in the G2/M phase. Furthermore, USP7 can overcome resistance to taxane drugs by inducing PLK1 protein degradation, potentially leading to chromosome misalignment during mitosis [44]. USP7 further enhances the chemoresistance of triple-negative breast cancer (TNBC) by deubiquitinating and stabilizing the ABCB1 protein [45]. 

In conclusion, the expression of USP7 has significant effects on cancer. A deeper study of its regulatory mechanisms and role in cancer could pave the way for new insights and therapeutic strategies for cancer treatment. Inhibiting USP7 expression or activity could potentially lead to better treatment outcomes and survival prognosis for cancer patients (Table 1: the expression of USP7 in cancer).

## 3. The Role of USP7 in Tumor Inflammation

USP7 plays a central role in tumor inflammation. It modulates the activity of target proteins through its deubiquitinating function, which includes proteins closely linked to tumorigenesis and progression. For instance, it can affect the stability of the tumor suppressor protein p53, thereby affecting its role in cell cycle regulation and apoptosis [44]. In the context of tumor inflammation, such regulation could lead to abnormal proliferation and survival of tumor cells, thereby increasing the inflammatory response. Second, USP7 plays a significant role in the immune response of tumors. Tumor inflammation typically results in infiltration of immune cells and activation of immune reactions. USP7 can influence the development and progression of tumor inflammation by affecting the function and activity of immune cells [57]. In addition, USP7 is also involved in regulating the metabolism and energy balance of tumor cells, which is an important aspect of the tumor inflammatory responses. The rapid growth and proliferation of tumor cells require a large amount of energy and material support, and USP7 can create favorable conditions for the growth of tumor cells by affecting the associated metabolic pathways [58]. In conclusion, it is worth noting that the overexpression or mutation of USP7 may be closely related to the occurrence and development of tumors [59,60]. Therefore, regulating the expression and activity of USP7 in tumor inflammatory responses could be a potential therapeutic strategy.

### 3.1. Initiation of Inflammatory Tumor Microenvironment

**Figure 4:**
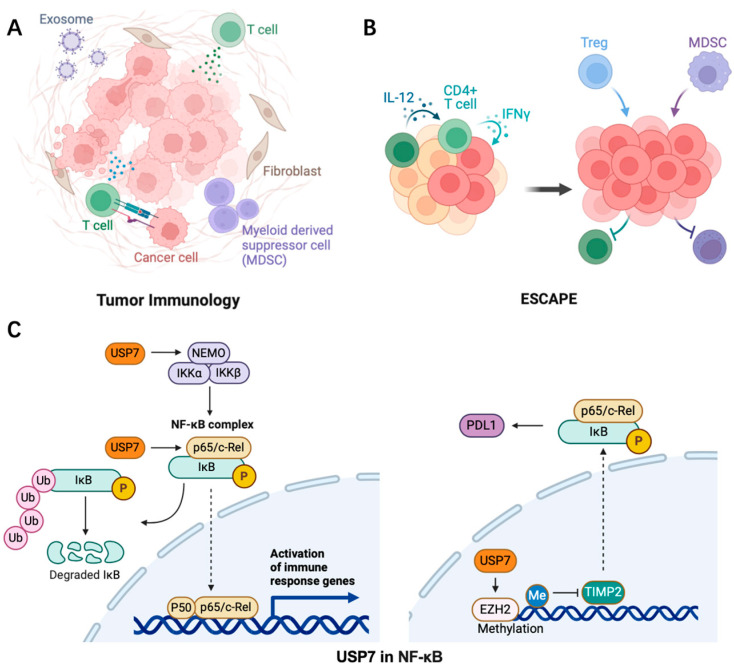
The role of USP7 (**C**) in promoting tumor inflammation (**A**) and inhibiting anti-tumor immunity (**B**) in the tumor microenvironment via NF-κB signaling. The dashed line represents the process that occurs simultaneously.

In cancer, the tumor microenvironment (TME) is a complex network composed of multiple cell types, including tumor cells, immune cells, and stromal cells. These cells influence tumor growth, metastasis, and treatment response through interactions (Figure 4). Inflammation contributes to tumor growth by supplying the tumor microenvironment with various bioactive components that promote proliferation, angiogenesis, invasion, and metastasis. The effectiveness of conventional cancer therapies is influenced by the inflammatory tumor microenvironment, which is characterized by interactions between local cells and immune elements [61,62]. USP7 maintains the survival and function of Tregs through ubiquitination and stabilization of Foxp3, an important transcription factor of Tregs. USP7 inhibitors can reduce the immunosuppressive function of Tregs and thereby enhance the anti-tumor immune response [63]. USP7 can also promote the degradation of MDM2 to inhibit the p53-mediated anti-tumor immune response. Inflammatory factors such as cytokines, chemokines, growth factors, and signaling pathways including NF-κB, JAK-STAT, TLR, cGAS/STING, and MAPK regulate the initiation and resolution of inflammation. Prostaglandins, leukotrienes, thromboxane, and specialized pro-resolving mediators (SPMs) are examples of inflammatory metabolites identified in inflammation [64].

At the molecular level, the initiation of tumor inflammation typically involves the activation of non-specific immune responses. The host organism recognizes and eliminates pathogens such as bacteria, viruses, fungi, and antigenic substances released by tumor cells through these non-specific immune responses [65]. During this process, a series of immune cells and molecules, including macrophages, natural killer cells, and the complement system, are activated [66,67]. Subsequently, the specific immune system is further activated, releasing a variety of inflammatory mediators like cytokines and chemokines. These mediators play a pivotal role in the inflammatory response by promoting the proliferation, differentiation, and migration of immune cells, and by modulating the activity of immune cells, thereby instigating and sustaining the inflammatory response [68]. In the tumor microenvironment, USP7 plays a significant role in the reprogramming of tumor-associated macrophages (TAMs). Studies have shown that USP7 is highly expressed in M2-type macrophages, and its inhibition can promote the transition from M2 to M1 type, thereby enhancing the anti-tumor immune response [4]. A recent study has found that USP7 and USP47 are involved in the activation of inflammasomes in macrophages and play a direct role in the deubiquitination of NLRP3 [69]. In addition, USP7 inhibitors can suppress tumor angiogenesis and increase the effectiveness of immune checkpoint inhibitors by downregulating VEGF secretion in fibroblasts [57].

Among cytokines, tumor necrosis factor-α (TNF-α) and the interleukin (IL) family hold a particularly significant status. They can activate the production of various inflammatory mediators and induce the activation of a variety of immune cells, thereby participating in the regulation of tumor inflammation [70]. According to the former study, it showed that through inhibiting the BiP-eIF2α-ATF4-CHOP signaling of ERS and NF-κB/p65 signaling, USP7 promotes chondrocyte proliferation and suppresses the apoptosis and inflammatory response under TNF-α-induced inflammation [71]. Additionally, chemokines play a crucial role in tumor inflammation. They guide immune cells towards the site of inflammation, promoting the local aggregation of immune cells within tumor tissues. This aggregation enhances the immune cells’ ability to attack and eliminate tumor cells [72]. However, tumor inflammation is not always beneficial to the host. Excessive or persistent inflammation can lead to tissue damage and dysfunction, and may even promote tumor growth and metastasis. Therefore, regulating the balance of tumor inflammation is of paramount importance in cancer treatment.

### 3.2. USP7 Regulates NF-κB Signaling

The transcription factor NF-κB plays a crucial role in modulating inflammation-related cancer by controlling inflammatory responses. Its influence on tumor development and progression is attributed to the activation of excessive innate immunity and abnormal cell growth. However, NF-κB also exerts a direct anti-inflammatory effect by inhibiting inflammasomes, which are important limiting factors that prevent excessive production of proinflammatory cytokines through tight control of NF-κB levels in the nucleus via proteasomal degradation. A recent discovery has revealed that USP7 functions as a specific deubiquitinase for the p65 subunit of NF-κB and its activity depends on DNA binding [72]. Furthermore, in hepatocellular carcinoma (HCC), the adapter protein PROX1 can promote NF-κB signaling by recruiting USP7 to the p65-p50 complex and facilitating p65 deubiquitination [73]. Additionally, USP7 negatively regulates NEMO polyubiquitination and inhibits IKKβ phosphorylation in TNFα-activated cells in conjunction with HSCARG (NmrA-like family domain-containing protein 1). Moreover, USP7 activates the canonical NF-κB signaling pathway in myeloma patients through MEK2 stabilization, which regulates the PP1α/AKT axis [74,75] (Figure 4).

Chronic inflammation may promote tumorigenesis through EZH2-dependent transcriptional activation of IL-6/TNF and suppression of IFNGR1. EZH2 physically interacts (without being catalytically active) with RelA/RelB in ER-negative basal-like breast cancer cells, leading to the expression of NF-kB target genes, namely IL-6, IL-8, and CSCL-1. On the other hand, EZH2 silences NF-κB by methylating its promoters in ER-positive luminal-like breast cancer cells, preventing the expression of the NF-kB target gene. Given that USP7 is responsible for stabilizing EZH2 and ERα, it is interesting to consider how USP7 might regulate inflammation by exploiting EZH2 stabilization based on the presence or absence of ER in breast cancer [76,77,78]. In addition, as previously mentioned, the EZH2-HMGA1-USP7 complex activates the cGAS-STING pathway, stabilizes Cgas, and may promote inflammation, which in turn supports breast cancer metastasis, but this requires additional research related to inflammatory responses and tumor progression [79].

In summary, USP7 plays diverse roles in tumor inflammatory responses, including regulating the stability of tumor-related proteins, influencing immune responses, and regulating tumor cell metabolism. An in-depth exploration of the functions and mechanisms of USP7 will help us better understand the process of tumor inflammatory responses and provide new ideas and methods for tumor treatment.

## 4. Discussion

Our current understanding of the specific role and mechanism of USP7 in tumor inflammatory responses is relatively limited. Although existing studies suggest that USP7 may influence the occurrence and development of tumor inflammatory responses by regulating the expression of various inflammation-related factors, the specific molecular mechanisms and signaling pathways still need to be further explored. Furthermore, the role of USP7 may differ in different tumor types, which requires further experimental data. Second, current research on inhibitors of USP7 is relatively limited. Although existing studies suggest that inhibitors of USP7 can suppress the proliferation and survival of tumor cells, thereby inhibiting the occurrence and development of tumors, no highly efficient and safe inhibitors against USP7 have been developed at present. Therefore, future research needs to investigate the structure and function of USP7 in more detail to develop more effective inhibitors. Finally, future research also needs to further explore the possible application of USP7 in tumor treatment. For example, inhibitors of USP7 can improve the effectiveness of immunotherapy by regulating tumor inflammatory responses. In addition, USP7 could also become a potential target for tumor treatment by inhibiting the activity of USP7 to suppress the occurrence and development of tumors. In summary, the role and mechanism of USP7 in the tumor inflammatory response is a complex process involving multiple signaling pathways and molecular mechanisms. Future research needs to further explore the specific role mechanism of USP7 in tumor initiation and development and develop more effective inhibitors of USP7 to improve the effectiveness of tumor treatment. At the same time, future research also needs to explore the structure and function of USP7 more deeply to develop more effective inhibitors.

## Figures and Tables

**Table 1:** The expression and role of USP7 in different types of cancer.

Type of Cancer	Expression	Functions	References
Lung cancer	high expression	Promote proliferation, inhibit cell death, and promote glycolysis	[29,42,46]
Osteosarcoma	high expression	Promote metastasis	[30,47]
Oral squamous cell carcinoma	high expression	Promote proliferation, migration, and invasion; reduce apoptosis	[31]
Melanoma	high expression	Promote proliferation, migration, and invasion; reduce apoptosis and senescence	[33,48]
Liver cancer	high expression	Promote proliferation, migration, and invasion	[36,49,50]
Cervical cancer	high expression	Promote proliferation, migration, and invasion; enhance immune escape ability; inhibit cell death	[14,42,51]
Glioblastoma	high expression	Inhibit apoptosis; promote tumor growth and metastasis	[34,40,52]
Nasopharyngeal carcinoma	high expression	Promote tumor progression and cisplatin resistance	[41]
Prostate cancer	high expression	Inhibit cell death; promote proliferation	[42,43]
Breast cancer	high expression	Induce chemoresistance; promote tumor progression	[45,53,54]
Esophagus cancer	high expression	Promote tumor growth, EMT, and metastasis	[55,56]

## References

[1] Snyder N.A., Silva G.M. (2021). Deubiquitinating enzymes (DUBs): Regulation, homeostasis, and oxidative stress response. J. Biol. Chem..

[2] Pozhidaeva A., Bezsonova I. (2019). USP7: Structure, substrate specificity, and inhibition. DNA Repair.

[3] Qi S.M., Cheng G., Cheng X.D., Xu Z., Xu B., Zhang W.D., Qin J.J. (2020). Targeting USP7-Mediated Deubiquitination of MDM2/MDMX-p53 Pathway for Cancer Therapy: Are We There Yet?. Front. Cell Dev. Biol..

[4] Dai X., Lu L., Deng S., Meng J., Wan C., Huang J., Sun Y., Hu Y., Wu B., Wu G. (2020). USP7 targeting modulates anti-tumor immune response by reprogramming Tumor-associated Macrophages in Lung Cancer. Theranostics.

[5] Ying H., Zhang B., Cao G., Wang Y., Zhang X. (2023). Role for ubiquitin-specific protease 7 (USP7) in the treatment and the immune response to hepatocellular carcinoma: Potential mechanisms. Transl. Cancer Res..

[6] Zhang N., Wang F., Zhang G., Zhang Q., Liu Y., Wang Q., Elsharkawy M.S., Zheng M., Wen J., Zhao G. (2022). USP7 Promotes deubiquitination and stabilization of MyD88 to enhance immune responses. Front. Immunol..

[7] Dong X., Yang C., Luo Y., Dong W., Xu X., Wu Y., Wang J. (2022). USP7 Attenuates Endoplasmic Reticulum Stress and NF-κB Signaling to Modulate Chondrocyte Proliferation, Apoptosis, and Inflammatory Response under Inflammation. Oxidative Med. Cell. Longev..

[8] Cockram P.E., Kist M., Prakash S., Chen S.H., Wertz I.E., Vucic D. (2021). Ubiquitination in the regulation of inflammatory cell death and cancer. Cell Death Differ..

[9] Al-Eidan A., Wang Y., Skipp P., Ewing R.M. (2022). The USP7 protein interaction network and its roles in tumorigenesis. Genes Dis..

[10] Wang C., Zhu Y., Zhu X., Chen R., Zhang X., Lian N. (2023). USP7 regulates HMOX-1 via deubiquitination to suppress ferroptosis and ameliorate spinal cord injury in rats. Neurochem. Int..

[11] Park H.B., Baek K.H. (2023). Current and future directions of USP7 interactome in cancer study. Biochim. Biophys. Acta Rev. Cancer.

[12] Hu M., Gu L., Li M., Jeffrey P.D., Gu W., Shi Y. (2006). Structural basis of competitive recognition of p53 and MDM2 by HAUSP/USP7: Implications for the regulation of the p53-MDM2 pathway. PLoS Biol..

[13] Pozhidaeva A.K., Mohni K.N., Dhe-Paganon S., Arrowsmith C.H., Weller S.K., Korzhnev D.M., Bezsonova I. (2015). Structural Characterization of Interaction between Human Ubiquitin-specific Protease 7 and Immediate-Early Protein ICP0 of Herpes Simplex Virus-1. J. Biol. Chem..

[14] Li N., Geng F., Liang S.M., Qin X. (2022). USP7 inhibits TIMP2 by up-regulating the expression of EZH2 to activate the NF-κB/PD-L1 axis to promote the development of cervical cancer. Cell. Signal..

[15] Yi J., Li H., Chu B., Kon N., Hu X., Hu J., Xiong Y., Kaniskan H.U., Jin J., Gu W. (2023). Inhibition of USP7 induces p53-independent tumor growth suppression in triple-negative breast cancers by destabilizing FOXM1. Cell Death Differ..

[16] Korenev G., Yakukhnov S., Druk A., Golovina A., Chasov V., Mirgayazova R., Ivanov R., Bulatov E. (2022). USP7 Inhibitors in Cancer Immunotherapy: Current Status and Perspective. Cancers.

[17] Do H.A., Baek K.H. (2022). Protein phosphatase 2A regulated by USP7 is polyubiquitinated and polyneddylated. Oncol. Rep..

[18] Liu J., Zhou T., Dong X., Guo Q., Zheng L., Wang X., Zhang N., Li D., Ren L., Yi F. (2023). De-ubiquitination of SAMHD1 by USP7 promotes DNA damage repair to overcome oncogenic stress and affect chemotherapy sensitivity. Oncogene.

[19] Oliveira R.I., Guedes R.A., Salvador J.A.R. (2022). Highlights in USP7 inhibitors for cancer treatment. Front. Chem..

[20] Bojagora A., Saridakis V. (2020). USP7 manipulation by viral proteins. Virus Res..

[21] Qian J., Pentz K., Zhu Q., Wang Q., He J., Srivastava A.K., Wani A.A. (2015). USP7 modulates UV-induced PCNA monoubiquitination by regulating DNA polymerase eta stability. Oncogene.

[22] Kashiwaba S., Kanao R., Masuda Y., Kusumoto-Matsuo R., Hanaoka F., Masutani C. (2015). USP7 Is a Suppressor of PCNA Ubiquitination and Oxidative-Stress-Induced Mutagenesis in Human Cells. Cell Rep..

[23] Georges A., Coyaud E., Marcon E., Greenblatt J., Raught B., Frappier L. (2019). USP7 Regulates Cytokinesis through FBXO38 and KIF20B. Sci. Rep..

[24] Zhang X.W., Feng N., Liu Y.C., Guo Q., Wang J.K., Bai Y.Z., Ye X.M., Yang Z., Yang H., Liu Y. (2022). Neuroinflammation inhibition by small-molecule targeting USP7 noncatalytic domain for neurodegenerative disease therapy. Sci. Adv..

[25] Kumagai J., Kiuchi M., Kokubo K., Yagyu H., Nemoto M., Tsuji K., Nagahata K., Sasaki A., Hishiya T., Onoue M. (2023). The USP7-STAT3-granzyme-Par-1 axis regulates allergic inflammation by promoting differentiation of IL-5-producing Th2 cells. Proc. Natl. Acad. Sci. USA.

[26] Guo N.J., Wang B., Zhang Y., Kang H.Q., Nie H.Q., Feng M.K., Zhang X.Y., Zhao L.J., Wang N., Liu H.M. (2024). USP7 as an emerging therapeutic target: A key regulator of protein homeostasis. Int. J. Biol. Macromol..

[27] Nicklas S., Hillje A.L., Okawa S., Rudolph I.M., Collmann F.M., van Wuellen T., Del Sol A., Schwamborn J.C. (2019). A Complex of the Ubiquitin Ligase TRIM32 and the Deubiquitinase USP7 Balances the Level of C-Myc Ubiquitination and Thereby Determines Neural Stem Cell Fate Specification. Cell Death Differ..

[28] Chang Y.C., Lin K., Baxley R.M., Durrett W., Wang L., Stojkova O., Billmann M., Ward H., Myers C.L., Bielinsky A.K. (2023). RNF4 and USP7 cooperate in ubiquitin-regulated steps of DNA replication. Open Biol..

[29] Zhang C., Lu J., Zhang Q.W., Zhao W., Guo J.H., Liu S.L., Wu Y.L., Jiang B., Gao F.H. (2016). USP7 promotes cell proliferation through the stabilization of Ki-67 protein in non-small cell lung cancer cells. Int. J. Biochem. Cell Biol..

[30] Zeng Q., Li Z., Zhao X., Guo L., Yu C., Qin J., Zhang S., Zhang Y., Yang X. (2019). Ubiquitin-specific protease 7 promotes osteosarcoma cell metastasis by inducing epithelial-mesenchymal transition. Oncol. Rep..

[31] Yang X., Jin J., Yang J., Zhou L., Mi S., Qi G. (2021). Expression of Ubiquitin-specific protease 7 in oral squamous cell carcinoma promotes tumor cell proliferation and invasion. Genet. Mol. Biol..

[32] Cai J.B., Shi G.M., Dong Z.R., Ke A.W., Ma H.H., Gao Q., Shen Z.Z., Huang X.Y., Chen H., Yu D.D. (2015). Ubiquitin-Specific Protease 7 Accelerates P14(ARF) Degradation by Deubiquitinating Thyroid Hormone Receptor-Interacting Protein 12 and Promotes Hepatocellular Carcinoma Progression. Hepatology.

[33] Xiang M., Liang L., Kuang X., Xie Z., Liu J., Zhao S., Su J., Chen X., Liu H. (2021). Pharmacological inhibition of USP7 suppresses growth and metastasis of melanoma cells in vitro and in vivo. J. Cell Mol. Med..

[34] Yi L., Cui Y., Xu Q., Jiang Y. (2016). Stabilization of LSD1 by Deubiquitinating Enzyme USP7 Promotes Glioblastoma Cell Tumorigenesis and Metastasis through Suppression of the P53 Signaling Pathway. Oncol. Rep..

[35] Gao L., Zhu D., Wang Q., Bao Z., Yin S., Qiang H., Wieland H., Zhang J., Teichmann A., Jia J. (2021). Proteome Analysis of USP7 Substrates Revealed Its Role in Melanoma Through PI3K/Akt/FOXO and AMPK Pathways. Front. Oncol..

[36] Ye M., He J., Zhang J., Liu B., Liu X., Xie L., Wei M., Dong R., Li K., Ma D. (2021). USP7 promotes hepatoblastoma progression through activation of PI3K/AKT signaling pathway. Cancer Biomark..

[37] Yoshihara H., Fukushima T., Hakuno F., Saeki Y., Tanaka K., Ito A., Yoshida M., Iemura S., Natsume T., Asano T. (2012). Insulin/Insulin-like Growth Factor (IGF) Stimulation Abrogates an Association between a Deubiquitinating Enzyme USP7 and Insulin Receptor Substrates (IRSs) Followed by Proteasomal Degradation of IRSs. Biochem. Biophys. Res. Commun..

[38] Vogt M., Classen S., Krause A.K., Peter N.-J., Petersen C., Rothkamm K., Borgmann K., Meyer F. (2024). USP7 Deregulation Impairs S Phase Specific DNA Repair after Irradiation in Breast Cancer Cells. Biomedicines.

[39] Liu X., Lu R., Yang Q., He J., Huang C., Cao Y., Zhou Z., Huang J., Li L., Chen R. (2024). USP7 reduces the level of nuclear DICER, impairing DNA damage response and promoting cancer progression. Mol. Oncol..

[40] Pan T., Li X., Li Y., Tao Z., Yao H., Wu Y., Chen G., Zhang K., Zhou Y., Huang Y. (2021). USP7 inhibition induces apoptosis in glioblastoma by enhancing ubiquitination of ARF4. Cancer Cell Int..

[41] Zhang B., Li J., Wang Y., Liu X., Yang X., Liao Z., Deng S., Deng Y., Zhou Z., Tian Y. (2024). Deubiquitinase USP7 stabilizes KDM5B and promotes tumor progression and cisplatin resistance in nasopharyngeal carcinoma through the ZBTB16/TOP2A axis. Cell Death Differ..

[42] Shin S.B., Kim C.H., Jang H.R., Yim H. (2020). Combination of Inhibitors of USP7 and PLK1 has a Strong Synergism against Paclitaxel Resistance. Int. J. Mol. Sci..

[43] Peng Y., Liu Y., Gao Y., Yuan B., Qi X., Fu Y., Zhu Q., Cao T., Zhang S., Yin L. (2019). USP7 is a novel Deubiquitinase sustaining PLK1 protein stability and regulating chromosome alignment in mitosis. J. Exp. Clin. Cancer Res..

[44] Xue Q., Yang D., Zhang J., Gan P., Lin C., Lu Y., Zhang W., Zhang L., Guang X. (2021). USP7, negatively regulated by miR-409-5p, aggravates hypoxia-induced cardiomyocyte injury. APMIS.

[45] Lin Y.-T., Lin J., Liu Y.-E., Chen Y.-C., Liu S.-T., Hsu K.-W., Chen D.-R., Wu H.-T. (2022). USP7 Induces Chemoresistance in Triple-Negative Breast Cancer via Deubiquitination and Stabilization of ABCB1. Cells.

[46] He Y., Jiang S., Zhong Y., Wang X., Cui Y., Liang J., Sun Y., Zhu Z., Huang Z., Mao X. (2023). USP7 promotes non-small-cell lung cancer cell glycolysis and survival by stabilizing and activating c-Abl. Clin. Transl. Med..

[47] Yang Z., Yu W., Xu A., Liu B., Jin L., Tao H., Wang D. (2024). mTORC1 accelerates osteosarcoma progression via m(6)A-dependent stabilization of USP7 mRNA. Cell Death Discov..

[48] Granieri L., Marocchi F., Melixetian M., Mohammadi N., Nicoli P., Cuomo A., Bonaldi T., Confalonieri S., Pisati F., Giardina G. (2022). Targeting the USP7/RRM2 axis drives senescence and sensitizes melanoma cells to HDAC/LSD1 inhibitors. Cell Rep..

[49] Sakamoto T., Kuboki S., Furukawa K., Takayashiki T., Takano S., Yoshizumi A., Ohtsuka M. (2023). TRIM27-USP7 complex promotes tumour progression via STAT3 activation in human hepatocellular carcinoma. Liver Int..

[50] Bian S., Ni W., Zhu M., Zhang X., Qiang Y., Zhang J., Ni Z., Shen Y., Qiu S., Song Q. (2022). Flap endonuclease 1 Facilitated Hepatocellular Carcinoma Progression by Enhancing USP7/MDM2-mediated P53 Inactivation. Int. J. Biol. Sci..

[51] Su D., Ma S., Shan L., Wang Y., Wang Y., Cao C., Liu B., Yang C., Wang L., Tian S. (2018). Ubiquitin-specific protease 7 sustains DNA damage response and promotes cervical carcinogenesis. J. Clin. Investig..

[52] Li J., Zhang B., Feng Z., An D., Zhou Z., Wan C., Hu Y., Sun Y., Wang Y., Liu X. (2024). Stabilization of KPNB1 by deubiquitinase USP7 promotes glioblastoma progression through the YBX1-NLGN3 axis. J. Exp. Clin. Cancer Res..

[53] Hayal T.B., DoĞan A., Şişli H.B., Kiratli B., Şahin F. (2020). Ubiquitin-specific protease 7 downregulation suppresses breast cancer in vitro. Turk. J. Biol..

[54] Wang Q., Ma S., Song N., Li X., Liu L., Yang S., Ding X., Shan L., Zhou X., Su D. (2016). Stabilization of histone demethylase PHF8 by USP7 promotes breast carcinogenesis. J. Clin. Investig..

[55] Hu T., Zhang J., Sha B., Li M., Wang L., Zhang Y., Liu X., Dong Z., Liu Z., Li P. (2019). Targeting the overexpressed USP7 inhibits esophageal squamous cell carcinoma cell growth by inducing NOXA-mediated apoptosis. Mol. Carcinog..

[56] Gao A., Zhang M., Zhu S.Q., Zou S., Chen H., Li X., He C., Zhou L., Mei Y., Ding W. (2024). DNA polymerase iota promotes EMT and metastasis of esophageal squamous cell carcinoma by interacting with USP7 to stabilize HIF-1α. Cell Death Dis..

[57] Jurisic A., Sung P.J., Wappett M., Daubriac J., Lobb I.T., Kung W.W., Crawford N., Page N., Cassidy E., Feutren-Burton S. (2024). USP7 inhibitors suppress tumour neoangiogenesis and promote synergy with immune checkpoint inhibitors by downregulating fibroblast VEGF. Clin. Transl. Med..

[58] Zhao X., Fu J., Hu B., Chen L., Wang J., Fang J., Ge C., Lin H., Pan K., Fu L. (2021). Serine Metabolism Regulates YAP Activity Through USP7 in Colon Cancer. Front. Cell Dev. Biol..

[59] Pei Y., Fu J., Shi Y., Zhang M., Luo G., Luo X., Song N., Mi T., Yang Y., Li J. (2022). Discovery of a Potent and Selective Degrader for USP7. Angew. Chem. Int. Ed. Engl..

[60] Valles G.J., Bezsonova I., Woodgate R., Ashton N.W. (2020). USP7 Is a Master Regulator of Genome Stability. Front. Cell Dev. Biol..

[61] Ma Y., Adjemian S., Mattarollo S.R., Yamazaki T., Aymeric L., Yang H., Portela Catani J.P., Hannani D., Duret H., Steegh K. (2013). Anticancer chemotherapy-induced intratumoral recruitment and differentiation of antigen-presenting cells. Immunity.

[62] Crusz S.M., Balkwill F.R. (2015). Inflammation and cancer: Advances and new agents. Nat. Rev. Clin. Oncol..

[63] Van Loosdregt J., Fleskens V., Fu J., Brenkman A.B., Bekker C.P.J., Pals C.E.G.M., Meerding J., Berkers C.R., Barbi J., Gröne A. (2013). Stabilization of the Transcription Factor Foxp3 by the Deubiquitinase USP7 Increases Treg-Cell-Suppressive Capacity. Immunity.

[64] Zhao H., Wu L., Yan G., Chen Y., Zhou M., Wu Y., Li Y. (2021). Inflammation and tumor progression: Signaling pathways and targeted intervention. Signal Transduct. Target. Ther..

[65] De Marco Castro E., Calder P.C., Roche H.M. (2021). β-1,3/1,6-Glucans and Immunity: State of the Art and Future Directions. Mol. Nutr. Food Res..

[66] Chen J., Gao L., Wu X., Fan Y., Liu M., Peng L., Song J., Li B., Liu A., Bao F. (2023). BCG-induced trained immunity: History, mechanisms and potential applications. J. Transl. Med..

[67] Burger E. (2021). Paracoccidioidomycosis Protective Immunity. J. Fungi.

[68] Abramiuk M., Grywalska E., Małkowska P., Sierawska O., Hrynkiewicz R., Niedźwiedzka-Rystwej P. (2022). The Role of the Immune System in the Development of Endometriosis. Cells.

[69] Palazón-Riquelme P., Worboys J.D., Green J., Valera A., Martín-Sánchez F., Pellegrini C., Brough D., López-Castejón G. (2018). USP7 and USP47 Deubiquitinases Regulate NLRP3 Inflammasome Activation. EMBO Rep..

[70] Boyman O., Sprent J. (2012). The role of interleukin-2 during homeostasis and activation of the immune system. Nat. Rev. Immunol..

[71] Griffith J.W., Sokol C.L., Luster A.D. (2014). Chemokines and chemokine receptors: Positioning cells for host defense and immunity. Annu. Rev. Immunol..

[72] Colleran A., Collins P.E., O’Carroll C., Ahmed A., Mao X., McManus B., Kiely P.A., Burstein E., Carmody R.J. (2013). Deubiquitination of NF-κB by Ubiquitin-Specific Protease-7 promotes transcription. Proc. Natl. Acad. Sci. USA.

[73] Liu Y., Zhang Y., Wang S., Dong Q.Z., Shen Z., Wang W., Tao S., Gu C., Liu J., Xie Y. (2017). Prospero-related homeobox 1 drives angiogenesis of hepatocellular carcinoma through selectively activating interleukin-8 expression. Hepatology.

[74] Gan Q., Li T., Hu B., Lian M., Zheng X. (2009). HSCARG inhibits activation of NF-kappaB by interacting with IkappaB kinase-beta. J. Cell Sci..

[75] Li T., Guan J., Li S., Zhang X., Zheng X. (2014). HSCARG downregulates NF-κB signaling by interacting with USP7 and inhibiting NEMO ubiquitination. Cell Death Dis..

[76] Xia X., Liao Y., Huang C., Liu Y., He J., Shao Z., Jiang L., Dou Q.P., Liu J., Huang H. (2019). Deubiquitination and stabilization of estrogen receptor α by ubiquitin-specific protease 7 promotes breast tumorigenesis. Cancer Lett..

[77] Lee S.T., Li Z., Wu Z., Aau M., Guan P., Karuturi R.K., Liou Y.C., Yu Q. (2011). Context-specific regulation of NF-κB target gene expression by EZH2 in breast cancers. Mol. Cell.

[78] Su D., Wang W., Hou Y., Wang L., Yi X., Cao C., Wang Y., Gao H., Wang Y., Yang C. (2021). Bimodal regulation of the PRC2 complex by USP7 underlies tumorigenesis. Nucleic Acids Res..

[79] Duan D., Shang M., Han Y., Liu J., Liu J., Kong S.H., Hou J., Huang B., Lu J., Zhang Y. (2022). EZH2-CCF-cGAS Axis Promotes Breast Cancer Metastasis. Int. J. Mol. Sci..

